# Low-temperature dechlorination of polyvinyl chloride (PVC) for production of H_2_ and carbon materials using liquid metal catalysts

**DOI:** 10.1126/sciadv.adm9963

**Published:** 2024-07-24

**Authors:** Felipe Polo-Garzon, Zili Wu, Yuanyuan Li, Junyan Zhang, Xinbin Yu, Elena Toups, Eddie Lopez-Honorato, Joshua T. Damron, Jeffrey C. Foster, Yongqiang Cheng, Luke L. Daemen, Anibal J. Ramirez-Cuesta, Harry M. Meyer

**Affiliations:** ^1^ Chemical Sciences Division, Oak Ridge National Laboratory, Oak Ridge, TN 37831 (USA).; ^2^ Center for Nanophase Materials Sciences, Oak Ridge National Laboratory, Oak Ridge, TN 37831 (USA).; ^3^ University of New Orleans, New Orleans, LA 70148 (USA).; ^4^ Nuclear Energy and Fuel Cycle Division, Oak Ridge National Laboratory, Oak Ridge, TN 37831 (USA).; ^5^ Neutron Scattering Division, Oak Ridge National Laboratory, Oak Ridge, TN 37831 (USA).; ^6^ Neutron Technologies Division, Oak Ridge National Laboratory, Oak Ridge, TN 37831 (USA).

## Abstract

Polyvinyl chloride (PVC) is ubiquitous in everyday life; however, it is not recycled because it degrades uncontrollably into toxic products above 250°C. Therefore, it is of interest to controllably dechlorinate PVC at mild temperatures to generate narrowly distributed carbon materials. We present a catalytic route to dechlorinate PVC (~90% reduction of Cl content) at mild temperature (200°C) to produce gas H_2_ (with negligible coproduction of corrosive gas HCl) and carbon materials using Ga as a liquid metal (LM) catalyst. A LM was used to promote intimate contact between PVC and the catalytic sites. During dechlorination of PVC, Cl is sequestrated in the carbonaceous solid product. Later, chlorine is easily removed with an acetone wash at room temperature. The Ga LM catalyst is reusable, outperforms a traditional supported metal catalyst, and successfully converts (untreated) discarded PVC pipe.

## Teaser

Ga liquid-metal catalyst dechlorinates PVC into carbon materials at low temperature while producing valuable H_2_.

## INTRODUCTION

Reincorporation of discarded plastics into the economy is key to protect the environment while enjoying the versatility and stability of plastic products. Recycling plastics reduces their accumulation in the environment, the carbon footprint associated with their production, and the emissions of CO_2_ and noxious gases generated during the incineration of discarded plastics (*1*). Polyvinyl chloride (PVC) is ubiquitous in everyday life. It is present in construction materials, household items, and medical supplies, to cite a few (*2*). PVC accounts for approximately 9% of plastics produced worldwide (*3*); however, it is not recycled as it uncontrollably deconstructs upon thermal treatment, producing corrosive HCl, along with toxic chlorinated hydrocarbons (*4*–*8*). Thus, developing an energy-efficient process to controllably convert discarded PVC into useful chemicals has attracted massive interest.

Today, hydrothermal treatment of PVC (220° to 300°C) is one of the most studied processes to remove chlorine from PVC (*4*). Catalytic conversion offers the opportunity to lower the energetic requirements to drive the chemical transformation and tailor the reaction products. However, using PVC as a reactant presents a major hurdle: It is solid and thermally decomposes without melting. Therefore, traditional solid catalysts would provide limited interfacial contact. Nonetheless, processes have been developed using solid absorbents/catalysts to: pre-dechlorinate PVC before subsequent hydrogenation/hydrogenolysis to yield long-chain hydrocarbons (*8*–*9*), upcycle PVC into porous carbon with special physicochemical properties (*10*–*11*), produce benzene along with carbon material (*12*), or selectively synthesize organic chlorides and polyethylene-like molecules (*13*). However, recovering solid catalysts from solid products can be cumbersome. Recently, during co-upcycling of PVC and polyethylene terephthalate using ionic liquids, Cl was extracted from PVC to generate terephthalic acid and 1,2-dichloroethane. Yet, production of valuable products from PVC alone is still elusive (*14*). In general, traditional methods to convert PVC involve the use of solid absorbents/catalysts to trap chlorine, require cumbersome solid-solid separation of the spent absorbent/catalyst and the remaining dechlorinated PVC, require the use of solvents, or require cofeed of H_2_ at high pressures. To overcome these challenges and promote intimate solid PVC-catalyst contact, we envision using liquid metal (LM) catalysts. LMs are expected to provide a close contact with solid PVC because LMs have been used in LM-polymer composites for stretchable electronics. (*15*–*18*). In addition, stable LM/carbon interfaces have been reported (*19*–*21*).

LM catalysts are metals in the liquid phase that have recently gained attention due to their coke resistance and enhanced catalytic properties. LMs have been used for alkane dehydrogenation (*22*–*24*), electrolysis of NaCl to produce Cl_2_ (in the industrial Chlor-alkali process) (*25*–*26*), methane reforming (*27*), coal liquefaction (*28*), propylene production from long-chain hydrocarbons (*29*), methane pyrolysis to produce solid carbon and H_2_ (*30*–*35*), and CO_2_ conversion to solid carbon (*36*–*37*). Prominent LMs have melting points as low as −19° to 271°C (*38*).

Uncontrolled thermal decomposition of PVC starts around 250°C (*6*). Therefore, the temperature used for controlled catalytic conversion of PVC must be below ~250°C, and the lower the operating temperature, the more energy-efficient would be the catalytic process. Thus, Ga-based catalysts were chosen as Ga has one of the lowest melting points (30°C) compared to other metals (In, 157°C; Sn, 232°C; Bi, 271°C; Pb, 327°C) and lower toxicity (Hg, melting point: −39°C). In addition, Ga has been proven to dissociate the C═O bond in CO_2_ at less than 200°C (*36*). The binding energy of C═O is greater than that of C─H, C─C, and C─Cl bonds.

In the present work, we tested LM catalysts of low melting point (Ga, In-Ga) to promote an intimate liquid-solid contact with the solid PVC reactant at mild temperatures. When the catalytic conversion was performed with the Ga LM catalyst, ~90% of chlorine in PVC was removed at 200°C while producing gaseous H_2_ (representing ~11% of hydrogen contained in PVC) and preventing the production of undesired gas phase HCl. Instead, chlorine was trapped in the solid carbonaceous product during reaction, and then, in a postreaction step, it was easily removed with an acetone wash. After reaction, the carbonaceous product was easily scooped from the LM. Next, the LM was solidified by cooling it down for recovery and reuse. This approach to controllably dechlorinate PVC at mild temperatures opens opportunities for PVC upcycling. It does not require elevated pressures, H_2_ cofeed, or solvents. In addition, Cl^−^ is trapped in the liquid phase, preventing release of corrosive and toxic HCl gas. Table S1 summarizes the advantages of the present process in comparison with the state of the art for PVC conversion.

## RESULTS

### Catalytic performance

**Fig. 1.**
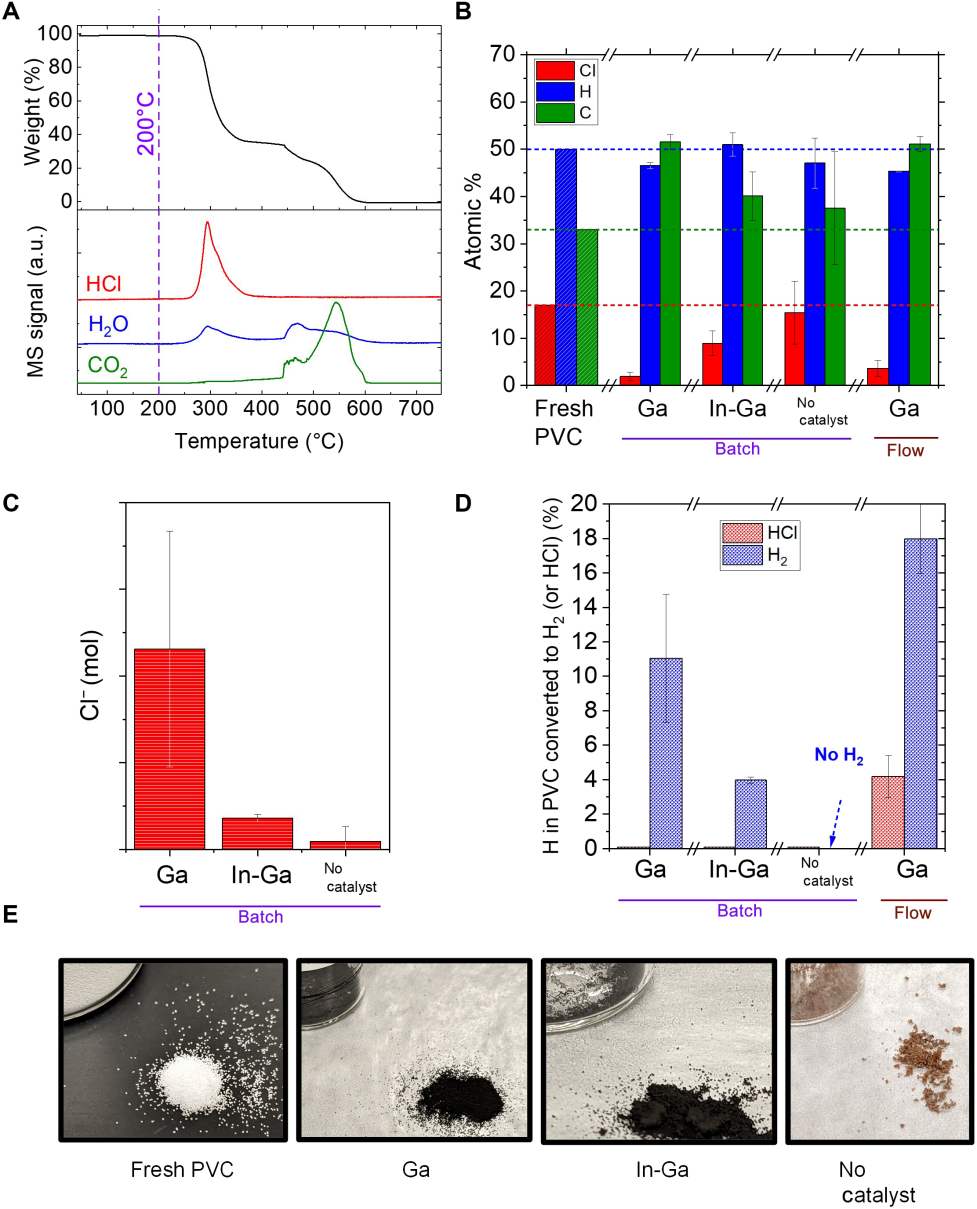
Characterization of PVC, carbon product, post-reaction wash, and gas overhead. (**A**) TGA-MS of fresh PVC in air. (B to E) Reaction was conducted under Ar atmosphere at 200°C for 1 hour using 5 g of LM and 0.2 g of PVC. In batch mode: Ar atmosphere, initial pressure: 0 psi. In flow mode: constant flow of 50 ml/min Ar. (**B**) Atomic composition of fresh PVC and carbonaceous products. (**C**) Chlorine content in acetone wash after washing carbonaceous products from using different catalysts. (**D**) Gas phase product distribution. (**E**) Pictures of carbonaceous products analyzed in (B) (pictures taken next to a vial of 2.5 cm in diameter). a.u., arbitrary units.

**Fig. 2.**
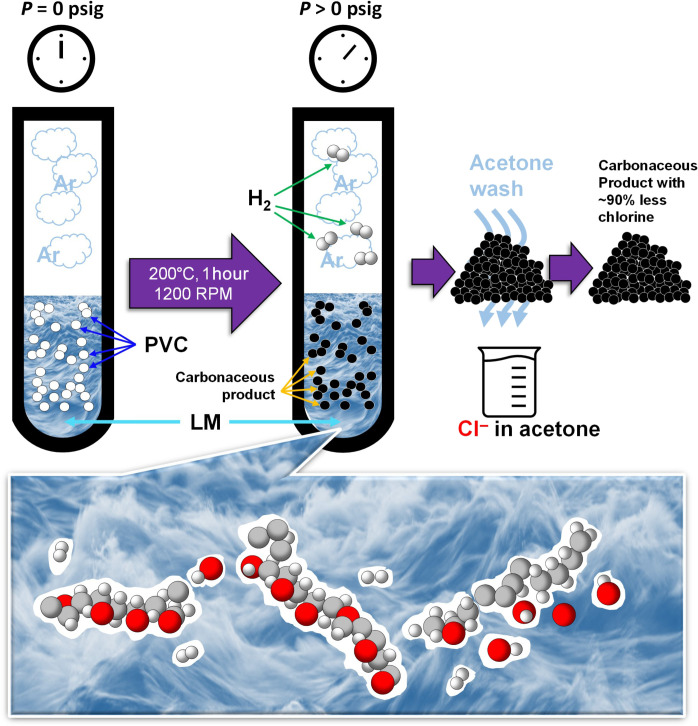
Conversion of PVC using LM catalysts (batch mode). Bottom sketch represents intimate PVC/LM contact.

Initially, the thermal decomposition of PVC in air was characterized via thermogravimetric analysis (TGA). The observed decomposition products were primarily HCl, H_2_O, and CO*_x_* (a small fraction of aromatics was detected but not included) as monitored via mass spectrometry (MS). Decomposition of PVC starts at 250°C; first, dechlorination and dehydrogenation take place; later, at around 450°C, the breaking of the C─C bond occurs (Fig. 1A). As negligible thermal decomposition occurs at 200°C, we tested the catalytic conversion of PVC using LM catalysts at this temperature. First, we performed catalytic batch conversion of 0.2 g of PVC in 5 g of LM (Ga or In-Ga) in a glass reactor. Before reaction, the reactor was flushed with Ar to evacuate air. After Ar flush, the inlet and outlet of the reactor were closed to create an Ar atmosphere at 0 psig. The reactor was introduced into an oil bath at 200°C, and the reaction was allowed to proceed for 1 hour under continuous stirring at 1200 rpm. After 1 hour of reaction, the reactor was removed from the oil bath and allowed to cool down naturally. The pressure inside the reactor was monitored using a pressure gauge. After the reactor cooled down to room temperature, the gas overhead was sampled through a septum and analyzed via MS. Later, the carbonaceous product was scooped from the LM (Fig. 2).

The carbonaceous product was washed in acetone to remove chlorine trapped in it. The final carbonaceous product, however, retained some Ga or In-Ga metal per TGA and energy-dispersive x-ray spectroscopy (EDS) characterization (fig. S1). Optimizing the acetone washing setup to fully remove LM leftover in the carbonaceous products lies beyond the scope of this work. As shown in Fig. 1B, catalytic conversion of PVC with Ga markedly reduced the concentration of Cl [from 17 to 2 atomic % (at %), representing a reduction in chlorine content of 88%], per analysis of the carbonaceous products via TGA-MS (see Materials and Methods section). To test whether the elemental composition of the LM provided special catalytic properties, we tested the eutectic In-Ga mixture (24.5% In, 75.5% Ga) as a catalyst for PVC conversion. The Cl content in the carbonaceous product using In-Ga (9 at %) was not as low as with Ga (2 at %). When PVC was treated in the glass reactor with the same reaction conditions but without a catalyst, the chlorine content remained nearly the same as for the fresh PVC. By monitoring the weight versus temperature profiles through TGA-MS of fresh PVC and carbonaceous products (fig. S2), one can qualitatively observe that the uncatalyzed conversion led to a carbonaceous product similar to fresh PVC. In contrast, the catalyzed conversion led to carbonaceous products of different weight versus temperature profiles.

The chlorine content obtained upon titration of the acetone wash revealed consistent trends with the chlorine content in the carbonaceous products (Fig. 1C). The less chlorine in the carbonaceous product, the more chlorine in the acetone wash. The catalytic conversion not only dechlorinated PVC but also promoted the production of gas H_2_, as shown in the analysis of the gas overhead (Fig. 1D). In batch operation, using Ga as catalyst, instead of In-Ga, led to greater formation of H_2_ (11% of H in PVC converted into gas phase H_2_ when Ga was used versus 4% when In-Ga was used). In flow operation, where the reactor head space was continuously flushed with Ar, using Ga as a catalyst, the production of H_2_ from PVC was higher than for batch operation using Ga. However, a substantial amount of gas phase HCl was coproduced, which is undesired for practical applications. The batch reaction at 150°C using Ga led to minor dechlorination of PVC (fig. S3). Therefore, we focus on batch conversion of PVC using Ga LM at 200°C.

### Characterization of carbonaceous products

**Fig. 3.**
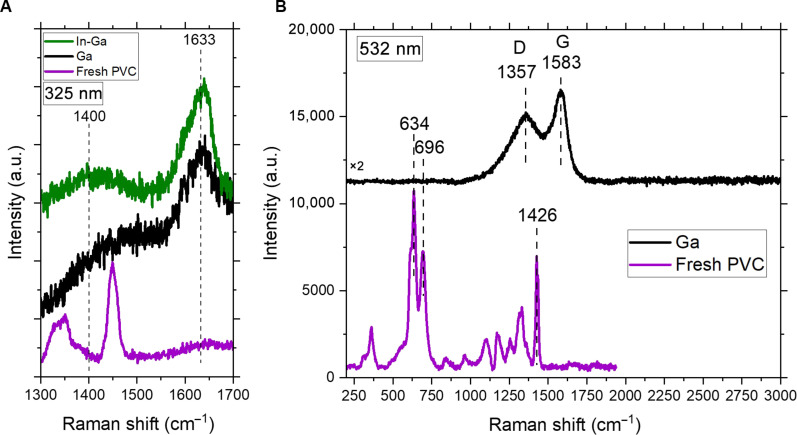
Characterization via Raman spectroscopy. (**A**) Raman spectroscopy (325-nm laser excitation) and (**B**) confocal Raman spectroscopy (532-nm laser excitation) of fresh PVC and carbonaceous product after batch reaction with Ga or In Ga LM catalysts at 200°C, 5 g of LM, 0.2 g of PVC, 1 hour, batch mode.

To study the chemical structure of the carbonaceous product, we performed Raman spectroscopy (Fig. 3 and fig. S4). As seen in fig. S4, in fresh PVC, bands at 637 and 700 cm^−1^ are attributed to stretching of the C─Cl bond. The bands at around 1450 and 2926 cm^−1^ are attributed to –CH_2_– deformation and stretching of C─H bonds in –CH_2_–, respectively (*39*–*40*). All of these bands for fresh PVC were essentially indistinguishable for the carbonaceous materials after reaction at 200°C with Ga or In-Ga LM catalysts (Fig. 3A and fig. S4A). Carbonaceous products obtained catalytically show Raman bands at around 1400 and 1633 cm^−1^ characteristic of carbon materials (*41*). Notably, the bands expected for conjugated double bond systems, 1109 and 1491 cm^−1^ (*41*), were not distinguishable in any of the carbonaceous products (Fig. 3A and fig. S4A). The vibrational features revealed by Raman spectroscopy are limited due to the low signal-to-noise ratio despite our attempts to optimize the experimental settings during data collection. Therefore, confocal Raman spectroscopy was used to take a closer look into the carbonaceous materials generated after the reaction with Ga LM catalyst at 200°C (Fig. 3B). Vibrational modes at 1357 and 1583 cm^−1^ are typical of D and G bands of graphitic carbon (*11*, *42*) The existence of the D band and the absence of a 2D b and (~2700 cm^−1^) reveal the amorphous nature of the carbon formed. The Raman spectrum of PVC treated at 200°C in the absence of a catalyst did not show substantial differences compared to the Raman spectrum of Fresh PVC (fig. S4B).

**Fig. 4.**
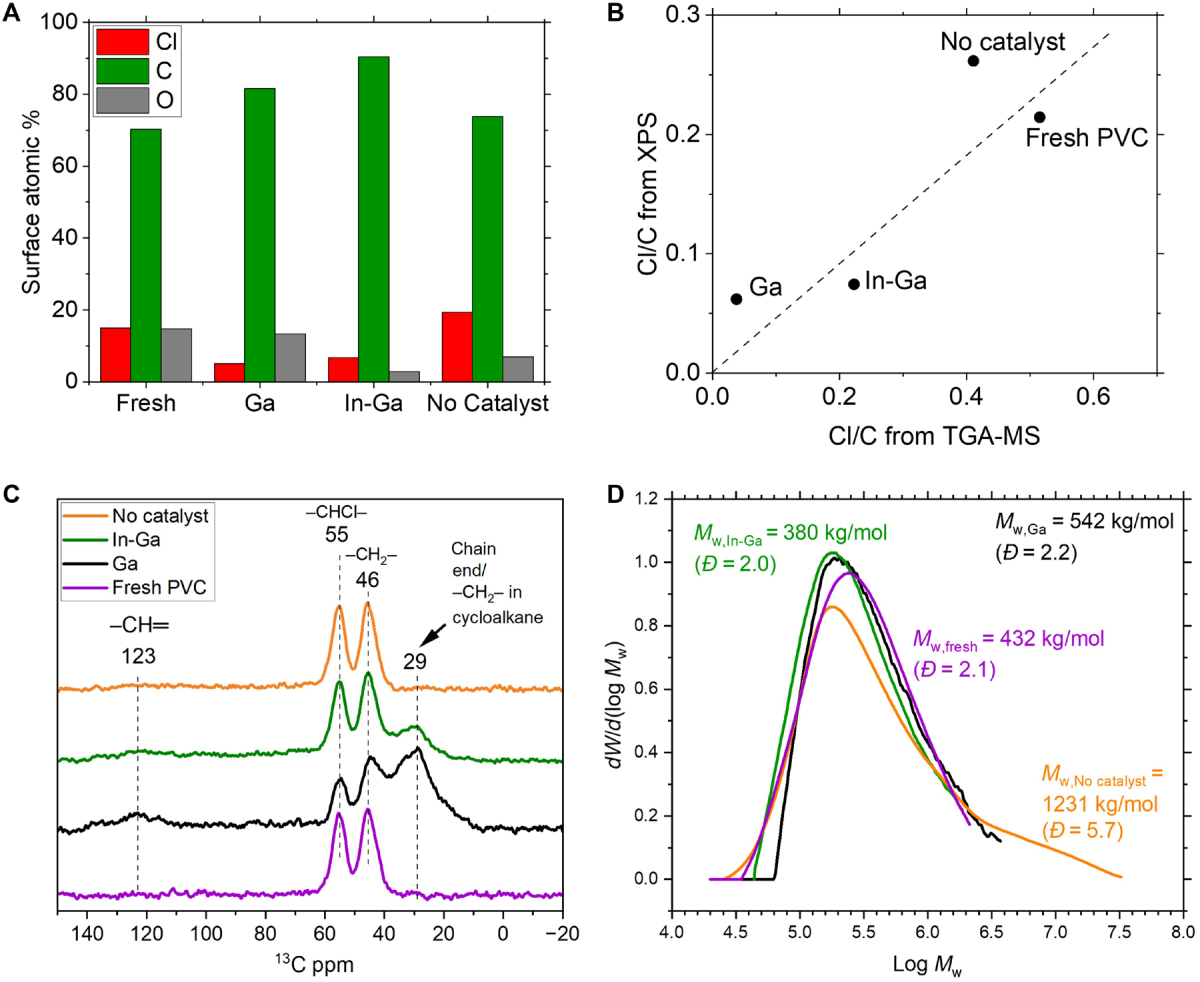
Characterization of surface composition, bonding structure, and molecular weight dsitribution. (**A**) Surface composition via XPS, (**B**) comparison of Cl/C ratio obtained via XPS and TGA-MS, (**C**) ^13^C MAS NMR and (**D**) SEC for fresh PVC and carbonaceous product after batch reaction with catalyst (“Ga,” “In-Ga”) and without catalyst (“no catalyst”) at 200°C, 5 g of LM, 0.2 g of PVC, 1 hour, batch mode.

To get insights into the carbon and chlorine bonds in the carbonaceous products after catalytic conversion with LM catalysts, we performed x-ray photoelectron spectroscopy (XPS). The surface composition of the carbonaceous products after catalytic conversion with Ga or In-Ga LMs and after thermal treatment (“No catalyst”) is presented in Fig. 4A. The carbonaceous products obtained through LM catalysis present lower Cl content compared with fresh PVC. In contrast, the uncatalyzed heat treatment did not lead to substantial dechlorination. Analysis of the surface composition via XPS [Fig. 4A and fig. S5 and adjacent discussion. XPS assignments are based on (*43*)] was congruent with the trends in C and Cl composition of the bulk (Fig. 1B), as shown in Fig. 4B. Further information on the chemical structure of the PVC reactant and the carbonaceous products (obtained with and without LM catalysts) was probed via infrared (IR) spectroscopy and ^13^C magic-angle spinning (MAS) nuclear magnetic resonance (NMR). IR spectroscopy showed the creation of a dechlorinated and more rigid structure when the conversion of PVC was catalyzed by LMs [fig. S6 and adjacent discussion. IR assignments are based on (*44*–*49*)]. The ^13^C-MAS NMR spectra (Fig. 4C) showed characteristic signatures for the PVC reactant, namely, the 55–and 46–parts per million (ppm) peaks that represent the methine (–CHCl–) and methylene carbons (–CH_2_–) of PVC, respectively (*50*–*51*). The carbonaceous product after catalysis with Ga had a lower relative concentration of methine carbon (bonded to Cl) than –CH_2_– carbons. This agrees with the chlorine reduction estimated via TGA-MS (Fig. 1B). Very minor signals in the aromatic region (120 to 150 ppm) were observed, only a weak peak observed at 123 ppm corresponding to olefinic carbon (─CH═). In contrast, a broad notable peak was observed centered around 29 ppm that could be assigned to a chain end (*52*–*53*) or carbon in a cycloalkane (cyclohexane = 27.6 ppm, cycloheptane = 28.2 ppm) (*54*). This suggests that the catalytic conversion led to more alkyl terminations and cycloalkanes, formed through crosslinking, rather than aromatic or polyenic compounds (*9*). Using In-Ga as the catalyst promoted minor dechlorination and some chain ends/cycloalkanes.

To gain insight into the molecular weight of the carbonaceous products obtained, size exclusion chromatography (SEC) was used (Fig. 4D). Before SEC experiments, the solid samples were stirred in tetrahydrofuran (THF) for at least 24 hours and filtered to remove insoluble material; however, only a minor fraction of the carbonaceous solids had dissolved during this process. While the SEC data do not provide information on the degree of chlorination of the soluble material, it is useful to identify general trends in the soluble carbonaceous material obtained. Catalytic conversion using Ga or In-Ga LMs led to minor changes in weight-average molecular weight (*M*_w_) (542 and 380 kg/mol for Ga and In-Ga, respectively) compared to the fresh PVC (432 kg/mol). In addition, the molecular weight distribution did not vary substantially before and after the catalytic conversion (*Đ* ~ 2). However, the molecular weight and dispersity following noncatalyzed heat treatment substantially increased (from *M*_w_ 432 to 1231 kg/mol and *Đ* from 2.1 to 5.7), and a second population of very high–molecular weight species could be observed in the molecular weight distribution. We attribute these changes to crosslinking mediated by thermally induced radical pathways, as has been demonstrated previously (*55*). Therefore, the SEC results in combination with the previous analyses [see ^13^C NMR (Fig. 4C)] show that the catalytic conversion successfully led to dechlorinated and cycloalkane-rich polymers while conserving the molecular weight distribution of the original sample. These data also highlight the limitations of thermal decomposition approaches (e.g., pyrolysis) in the absence of a catalyst.

**Fig. 5.**
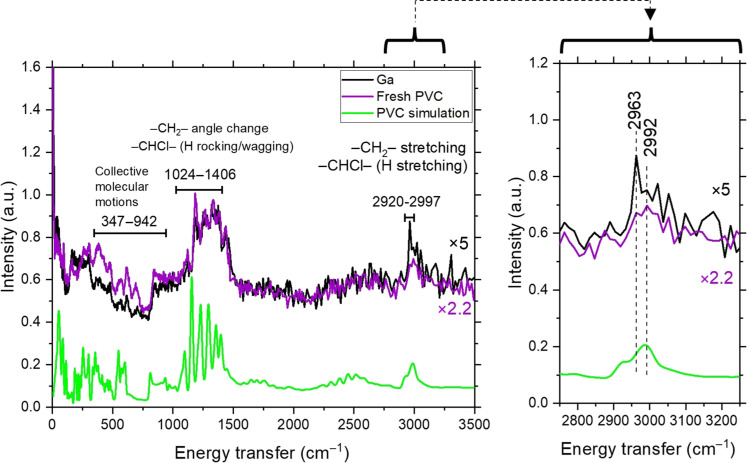
INS spectra. Fresh PVC and carbonaceous product after batch reaction with catalyst (Ga). Reaction conditions: 200°C, 5 g of LM, 0.2 g of PVC, 1 hour, batch mode.

To gain insight into the hydrogen species present in the carbonaceous product, we used inelastic neutron scattering (INS), which is extremely sensitive to vibrations of H-containing bonds (Fig. 5). In addition, INS is highly sensitive to collective molecular motions in the polymer chain. The spectral features of PVC and the carbonaceous product after catalytic conversion with Ga are similar. Yet, key differences can be spotted: Vibrations with energy transfer between 347 and 942 cm^−1^ for the carbonaceous product were suppressed when compared with pure PVC, vibrations with energy transfer between 1024 and 1406 cm^−1^ remain relatively unchanged, and at 2963 cm^−1^, the carbonaceous product presents an increased signal intensity. To identify the vibrational modes corresponding to the energy transfer observed, we simulated the spectrum for PVC using density functional theory (DFT). Vibrations of low energy transfer (347 to 942 cm^−1^) correspond to collective (nonlocalized) molecular motions in the polymer chain. Higher energy transfers (>1000 cm^−1^) are a combination of localized vibrational modes, as thoroughly listed in table S2. Nonetheless, general conclusions can be drawn. In the carbonaceous product, vibrational modes associated with collective motions are diminished, which agrees with the generation of more rigid cycloalkanes. However, this solid carbon product has an enhanced contribution from –CH_2_– asymmetrical stretching and H stretching in –CHCl– (~2963 to 2965 cm^−1^; table S2).

**Fig. 6.**
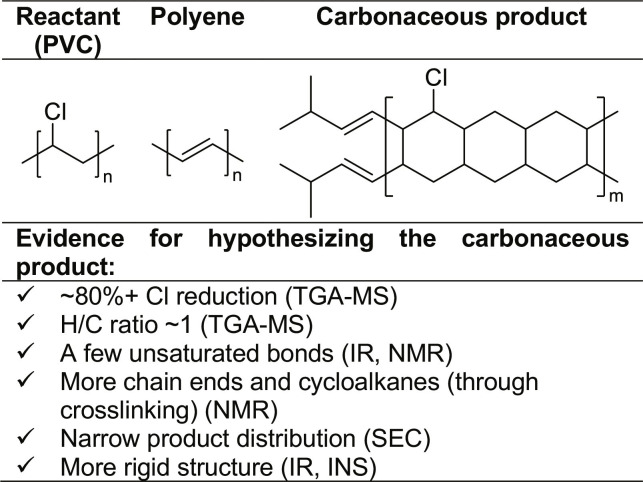
Sketch of plausible structure of the carbonaceous product (Ga LM, batch mode, 200°C). Polyene structure is included for comparison.

The characterization of the carbonaceous products from multiple complementary approaches allowed us to elucidate a plausible chemical structure, consisting of crosslinked polymers generating saturated cycloalkanes with more chain ends than the original PVC and with a minor concentration of unsaturated bonds (Fig. 6).

### LM-PVC interaction

To learn about the LM-PVC interactions, we performed XPS characterization of the fresh and spent Ga catalyst surface. Carbon and oxide overlayers on the fresh or spent Ga LM were observed. However, the presence of these overlayers does not compromise the catalytic process, as the fresh catalyst was used without further treatment (fig. S7). To study the bonding interactions between PVC and the Ga LM, we performed x-ray absorption spectroscopy (XAS) of the fresh LM catalyst (before reaction), spent LM catalyst (recovered after reaction), and the carbon product, for both Ga and In-Ga LMs (collection in fluorescence mode). For the Ga K edge data of fresh and spent Ga LM (and In-Ga LM), self-absorption effects most likely exist due to the high concentration of Ga in these samples. Thus, we only compare the rising edge positions and spectral shapes of Ga K edge XAS data rather than the peak intensities. For Ga remaining in the carbonaceous products obtained using Ga and In-Ga LMs and for In in fresh and spent In-Ga LM samples, due to their low concentrations, no self-absorption effects were observed.

**Fig. 7.**
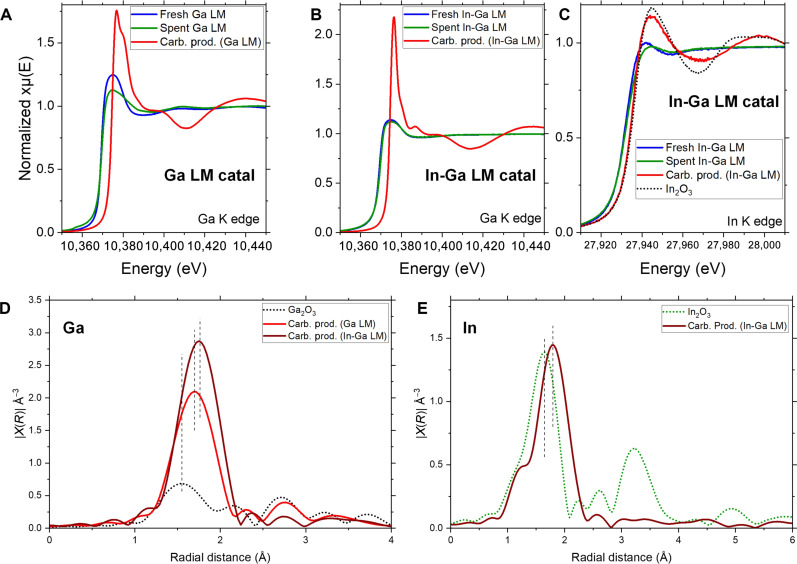
XAS spectra. XANES spectra. (**A**) Ga K edge of fresh Ga LM, spent Ga LM, and carbon product using Ga LM. (**B**) Ga K edge of fresh In-Ga LM, spent In-Ga LM, and carbon product using In-Ga LM. (**C**) In K edge of fresh In-Ga LM, spent In-Ga LM, and carbon product using In-Ga LM. [(D) and (E)] R space EXAFS spectra: (**D**) Ga embedded in the carbon product obtained using Ga or In-Ga LM catalysts, and Ga2O3 reference. (**E**) In embedded in the carbon product obtained using In-Ga LM catalyst and In_2_O_3_ reference. Reaction conditions: batch mode, Ar atmosphere, 200°C, 1 hour, 0.2 g of fresh PVC.

When Ga was used as LM catalyst, the fresh and spent LM consisted of mostly of metallic Ga; however, when analyzing the carbon product, the Ga embedded in it existed in an oxidized state as shown by x-ray absorption near edge structure (XANES) results (Fig. 7A) (*56*). Similarly, when In-Ga was used as LM catalyst, the In and Ga atoms embedded in the carbon product were in an oxidized state, whereas the fresh and spent LM were in the metallic state (Fig. 7, B and C) (*57*–*58*). The LMs present slight differences in their electronic and bonding structure before and after reaction. For instance, a Ga-Ga bond expansion in the Ga LM catalyst was observed after reaction (fig. S8 and table S3).

The electronic structure of Ga embedded in the carbon product is different depending on the LM catalyst used (Ga or In-Ga) (Fig. 7, A and B), suggesting that Ga has different local bonding environment in these two samples. Extended x-ray absorption fine structure (EXAFS) spectra for Ga embedded in the carbon product obtained using either LM catalyst show that the radial distance of the prominent peak is longer than the distance of the Ga─O bond in Ga_2_O_3_, and it is likely due to Ga─Cl contribution (Fig. 7D and table S3). Similarly, EXAFS spectra for In-Ga embedded in the carbon product suggest that the peak at 1.80 Å is due to the contribution of the In-Cl interaction (Fig. 7E and table S3). Clearly, the LM-PVC interaction alters the electronic and bonding structure of the LM metal. Although Ga─Cl and In─Cl bonds were formed when Ga and In-Ga catalysts were used, their performance for dechlorinating PVC and producing gas H_2_ was substantially different (Fig. 1). This suggests that both Ga and In-Ga LM catalyst can successfully activate PVC, but pure Ga is more efficient in the completion of the catalytic cycle.

### Scalability, catalyst reuse, and catalytic mechanism

**Fig. 8.**
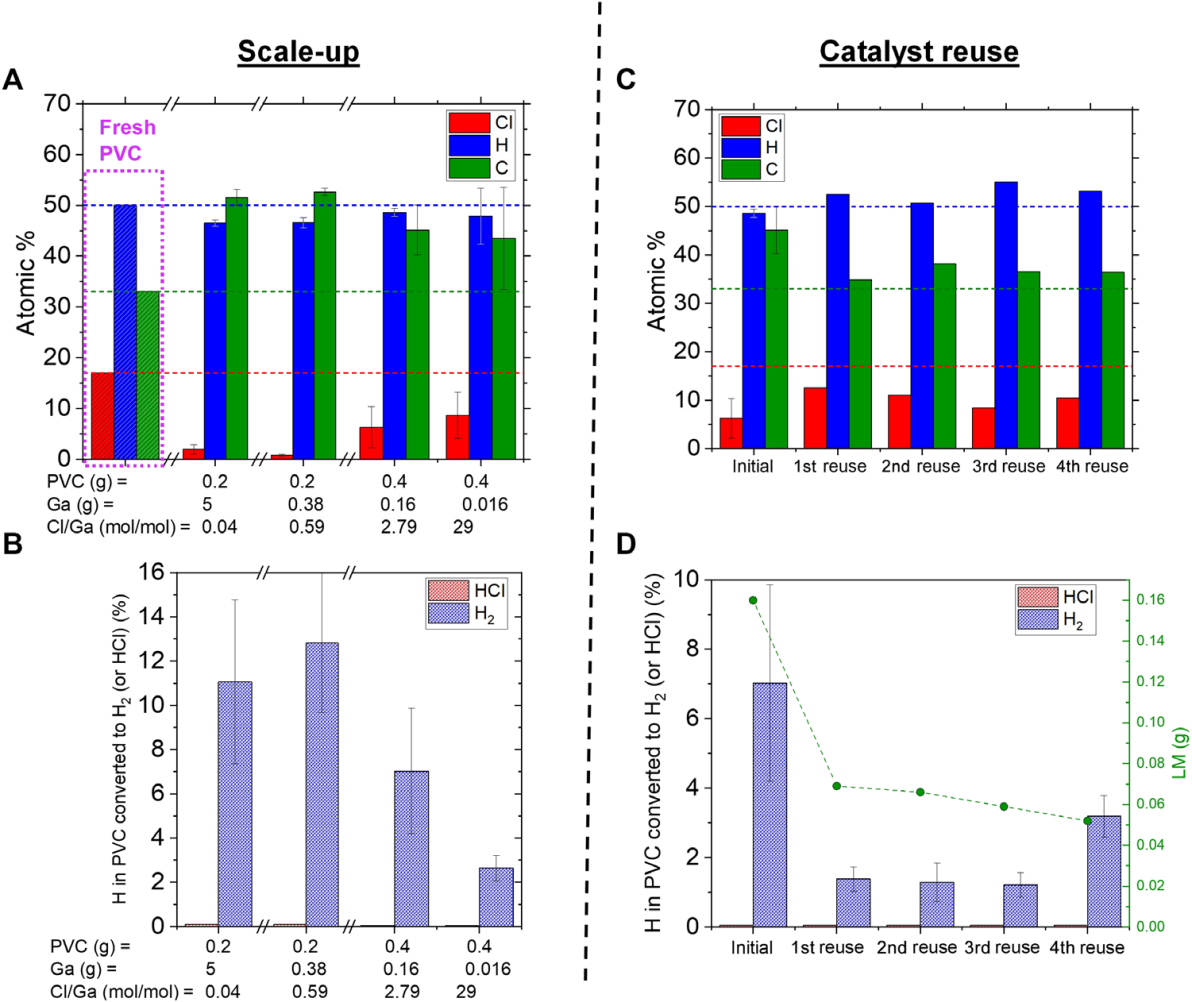
Scalability and catalyst reuse. (**A** and **B**) Test of scalability using various amounts of PVC and Ga LM catalyst: (A) atomic composition of carbonaceous products and (B) gas H_2_ and HCl product distribution. (**C** and **D**) Test of catalyst reuse with initial 0.16 g of Ga LM catalyst and a reload of 0.4 g of PVC for each cycle: (A) atomic composition of carbonaceous products and (B) gas H_2_ and HCl product distribution. Reaction was conducted in batch mode under Ar atmosphere at 200°C for 1 hour.

So far, catalytic tests have been performed with an excess amount of LM (5 g of LM, 0.2 g of PVC, molar ratio of Cl/LM = 0.04). Given that Ga─Cl bonds are created as characterized by XAS, it is reasonable to question whether the conversion is truly catalytic or if Ga acts as a sink for Cl^−^. To determine whether the conversion is truly catalytic, we performed the transformation with increased amount of PVC with respect to Ga (Fig. 8, A and B). When the Cl/LM molar ratio was increased to 0.59, the H_2_ yield and extent of dechlorination remained unchanged (Fig. 8, A and B, and fig. S9). As the Cl/Ga ratio was further increased, the extent of dechlorination dropped and the H_2_ yield. Despite the drop in H_2_ yield, the pressure of the overhead space exceeded the value expected from the concentration of H_2_ and HCl, suggesting that other volatiles were produced; nevertheless, they are not identified at the moment. At Cl/Ga molar ratio of 29, dechlorination of ~50% is attained, suggesting that the process is catalytic, and Ga does not act solely as a Cl^−^ sink (e.g., forming GaCl_3_).

To further confirm the catalytic nature of the process, we evaluated whether the Ga LM can be reused. After an initial run, the spent LM was separated, the adsorbed carbonaceous product was manually brushed away to the extent possible, and then the LM was immediately introduced into a reactor with fresh PVC. For these tests, an initial Cl/Ga molar ratio of 2.79 (0.4 g of PVC, 0.16 g of PVC) was used. If the transformation was stoichiometric (not catalytic) through formation of gallium chloride, then the dechlorination and H_2_ yield would decline notably with reuse. As observed in Fig. 8 (C and D), after four cycles, the extent of dechlorination of PVC (fresh PVC was loaded for each cycle) and the H_2_ yield remained relatively stable despite an initial decay in performance after the first cycle. The reusability of the catalyst was also confirmed using excess LM (Cl/Ga mol ratio of 0.04) (fig. S10, A and B), with negligible decay in performance.

Because supported metal catalysts have been shown to promote conversion (~250° to 300°C) of polyolefins (*59*–*61*), we tested a typical supported metal catalyst (Pt/Al_2_O_3_) for the conversion of PVC at the same reaction conditions as the Ga LM catalyst. Minor dechlorination of PVC and negligible production of H_2_ gas were observed (fig. S10, C and D), likely due to the poor PVC-catalyst interaction. In addition, the Pt/Al_2_O_3_ catalyst was hard to recover from the solid-solid post-reaction mixture, highlighting the practical advantages of using LM catalysts.

Last, a discarded piece of PVC pipe was gathered, and without any treatment, it was shredded into flakes. The flakes of PVC pipe were catalytically converted using Ga LM at 200°C (fig. S11). The Cl/H/C elemental composition of the PVC pipe resembled that of fresh PVC (commercially sourced powder) used so far in this work. The dechlorination of PVC pipe exceeded 90%, similar to the pure PVC powder (fig. S11A). In addition, H_2_ production was attained, without the presence of substantial gas phase HCl (fig. S11B). Negligible dechlorination of the PVC pipe and H_2_ production was observed when the transformation was attempted in the absence of the LM catalyst.

**Fig. 9.**
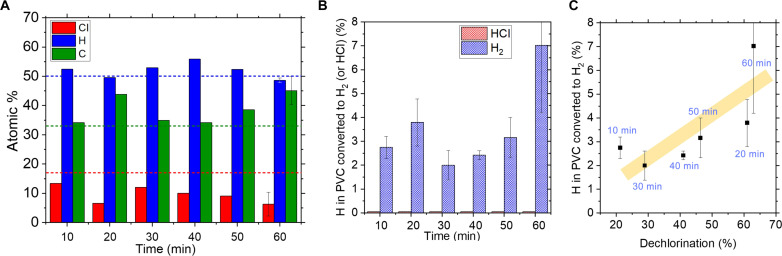
PVC conversion at different reaction times. (**A**) Composition of carbon product, (**B**) H_2_ and HCl yield, and (**C**) correlation between H_2_ yield and extent of dechlorination. Reaction was conducted in batch mode under Ar atmosphere at 200°C using 0.4 g of PVC and 0.16 g of Ga [Cl/Ga = 2.79 (mol/mol)].

**Fig. 10.**
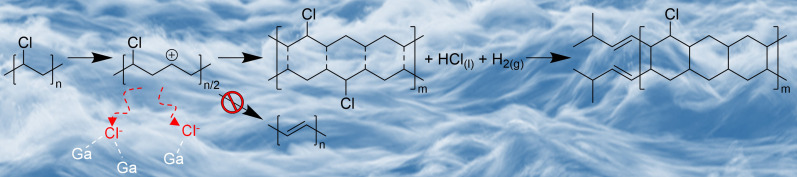
Proposed reaction mechanism. PVC upcycling to H_2_ and carbon materials using Ga LM catalyst.

To gain insights into the reaction mechanism occurring on the Ga LM catalyst, the reaction was conducted for different times and the products were analyzed (Fig. 9, A and B). The extent of dechlorination and H_2_ yield were found to be correlated (Fig. 9C) and to fluctuate up and down with reaction times varying from 10 to 60 min. This suggests that dechlorination of PVC and H_2_ production are concerted, and there is an equilibrium between the gas phase and the reactant mixture, so that H_2_ is reincorporated into the reacting species. The evidence collected allows to hypothesize a reaction mechanism where PVC is dehydrochlorinated by the Ga catalyst, but instead of leading to polyenes, the Ga catalyst favors crosslinking of the dechlorinated chains, leading to cyclic polymers that are mostly aliphatic. The identification of Ga─Cl bonds via XAS supports that the C─Cl bond in PVC activates on the catalyst surface (Fig. 10). At this stage, although we hypothesize that dechlorination of PVC and generation of H_2_ are concerted steps, the exact mechanism for H_2_ production remains unknown and requires further research.

## DISCUSSION

To the best of our knowledge, LM catalysts are used for the first time in this work to convert PVC into valuable chemicals. Because PVC does not melt but decomposes uncontrollably as temperature increases, using a LM catalyst was key to promote intimate contact with the reactant. When Ga LM catalyzes the conversion of PVC in Ar at 200°C, gas H_2_ is produced without coproducing corrosive gas HCl, along with a carbon material that traps Cl. After the reaction, Cl can be easily removed from the carbonaceous material at room temperature with an acetone wash. The carbon material has ~90% less Cl than the original PVC and consists of a more rigid crosslinked structure, likely cycloalkanes. In addition, the carbonaceous product has a low concentration of unsaturated bonds and more chain ends than the parent PVC. Further, the conversion catalyzed by Ga LM generated a product with narrow molecular weight distribution.

Tests show that the catalytic process is scalable, and the catalyst is reusable. In the absence of the Ga LM catalyst, no relevant PVC conversion was observed at 200°C. Further, our approach surpasses the performance of a typical solid supported noble-metal catalyst and facilitates catalyst/product separation after reaction. The catalytic conversion reported here was equally successful when a discarded PVC pipe was used as the reactant. Hinderance of HCl gas production during the conversion of PVC opens a gateway for coprocessing of poorly sorted plastic mixtures. The catalytic conversion presented here steered product selectivity away from polyenes and into polycyclic chains. This control of product selectivity motivates LM catalyst design to envision the production of value-added nanostructures.

## MATERIALS AND METHODS

### Materials

Gallium was purchased from Alfa Aesar (99.99%), and eutectic In-Ga (24.5% In, 75.5% Ga) was purchased from Sigma-Aldrich (99.99%). PVC was purchased from Sigma-Aldrich [average *M*_w_ ~233,000, average number-average molecular weight (*M*_n_) ~99,000] (referred to as “fresh PVC”). Phenolphthalein solution was purchased from Sigma-Aldrich [0.5 weight % in ethanol:water (1:1)]. A 5% Pt/Al_2_O_3_ was obtained from Johnson Matthey.

### Catalytic reaction

The reaction was conducted in a pressure-rated glass tube reactor of approximately 38 ml of volume and 20-cm long. A total of 0.2 to 0.4 g of PVC (either PVC powder sourced from Sigma-Aldrich sieved to 177 to 250 μm or flakes of discarded PVC pipe) were introduced in the reactor, along with the catalyst (0.016 to 5 g of LM or 0.4 g of Pt/Al_2_O_3_ sieved to 177 to 250 μm), and a stir bar of 1.1 to 1.5 cm in length. The reactor was then sealed and flushed with Ar (50 ml/min) to evacuate air. For batch mode operation, the Ar inlet and outlet were closed, and the reactor was placed in an oil bath preheated to 200°C. For the flow mode operation, Ar was kept flushing the system and the reactor was placed in an oil bath preheated to 200°C. The reactant mixture was stirred at 1200 rpm (9 to 12 relative centrifugal force) for all runs presented in the manuscript. The reaction was conducted for 10 to 60 min. After the reaction, the reactor was removed from the oil bath and allowed to cool down to room temperature naturally. For the batch mode, after cooling down to room temperature, the gas overhead was sampled by inserting a syringe needle through a septum. The gases were analyzed in a mass spectrometer (Pfeiffer). For the flow mode, the gases produced were continuously monitored using the MS. The signals monitored in the MS were Ar (*40*), HCl (*36*), Cl_2_ (*62*), H_2_ (*2*), H_2_O (*18*), CO_2_ (*44*), and other possible C_1_-C_2_ chlorinated hydrocarbons. The glass reactor was equipped with a pressure gauge and pressure relief valve.

After sampling the gas overhead, the carbonaceous product, spent LM, and stir bar were manually separated. The carbonaceous product was washed three times in acetone and centrifuged at 10,000 rpm. The carbonaceous product was separated and dried at 95°C overnight. The acetone wash was titrated for chlorine content.

The concentration of Cl, H, and C in the solid carbonaceous products was measured by comparing the MS signals during TGA of the carbonaceous product versus Fresh PVC. For simplicity, it was assumed that the only products of calcining PVC and carbonaceous products were HCl, H_2_O, and CO*_x_*. The Cl and H content in the solid carbonaceous products was found through calibration of the HCl and H_2_O signals. The C content was found assuming 100% material balance.

#### Thermogravimetric analysis–MS

TGA-MS was performed in a TA TGA Q5000 system coupled with a ThermoStar Pfeiffer mass spectrometer. In the TGA procedure, the sample was exposed to synthetic air (100 ml/min). The temperature was initially held at ~42°C for 30 min and then ramped up to 750°C at 10°C/min.

#### Energy-dispersive x-ray spectroscopy

EDS was performed in a Bruker Nano GmbH system using a XFlash detector 5030.

#### Titration using phenolphthalein

Titration using phenolphthalein was used to measure the chlorine content in the acetone wash. The acetone wash (after wash and centrifugation of the carbonaceous product) was mixed with phenolphthalein solution. An aqueous 0.1 M NaOH solution was loaded in a burette and added dropwise to the acetone + phenolphthalein solution while stirring at 700 rpm until the solution became pink, signaling that chlorine titration had finalized.

#### Raman spectroscopy

Raman spectrocopy was performed ex situ on a multiwavelength Raman system (*63*) at room temperature using 244- and 325-nm ultraviolet (UV) laser excitation. Raman scattering was collected via a customized ellipsoidal mirror and directed by a fiber optics bundle to the spectrograph stage of a triple Raman spectrometer (Princeton Instruments Acton TriVista 555). Edge filters (Semrock) were used in front of the UV-VIS fiber optic bundle (Princeton Instruments) to block the laser irradiation. The 325-nm excitation is generated from a HeCd laser (Melles Griot). A UV-enhanced liquid N_2_-cooled charge-coupled device detector (Princeton Instruments) was used for signal detection. The sample holder was held stationary, and the sample was analyzed for 10 s.

#### Confocal Raman spectroscopy

Confocal Raman spectroscopy was performed using a Renishaw InVia Qontor system using a 532 nm visible laser excitation. Spot analysis was performed using a 50x long distance work lenses.

#### X-ray photoelectron spectroscopy

XPS was performed ex situ using a Thermo Scientific (Waltham, MA, USA) Model K-Alpha XPS instrument. The instrument uses monochromated, micro-focused, Al Kα x-rays (1486.6 eV) with a variable spot size (i.e., 30 to 400 μm). A 400-μm x-ray spot size was used for sample analysis for maximum signal and to obtain an average surface composition over the largest possible area. The instrument has a hemispherical electron energy analyzer equipped with a 128-channel detector system. The analysis chamber has a pressure of approximately 2 × 10^−9^ mbar or lower.

A short Ar-ion depth profile was acquired for analyzing the fresh and spent Ga LM sample by alternately etching the surface with 2-kV Ar-ions and collecting core level spectra of elements of interest. After depth profiling, a set of survey and core level data were acquired on the well-etched surfaces of the samples.

#### Solid-state MAS NMR

Solid-state MAS NMR was performed on a 700-MHz VNMRs Varian NMR spectrometer with a triple resonance 3.2-mm probe. Direct detection experiments were performed using a Hahn echo refocusing over a single-rotor period spinning at 10 kHz.

#### Diffuse reflectance infrared Fourier transform spectroscopy

Diffuse reflectance infrared Fourier transform spectroscopy (DRIFTS) was performed with a Nicolet Nexus 670 FTIR spectrometer. Each sample was loaded into a crucible, which was placed in a DRIFTS cell (Pike Technologies). Each sample was pretreated in situ at 95°C under 30 ml/min of Ar for 1 hour. Next, the temperature was taken to 25°C. After 15 to 20 min of stabilization, a spectrum was collected.

#### Size exclusion chromatography

SEC analysis was performed on an Agilent 1260 Infinity II LC system equipped with an Agilent MiniMIX-C Guard column (PLGel 5 μM; 50 mm by 4.6 mm) and two Agilent MiniMIX-C columns (PLgel; 4.6 mm by 250 mm, 5 μm). The mobile phase used THF (HPLC grade) at flow rate of 0.3 ml/min [a set of narrow *M*_W_ polymethyl methacrylate (PMMA) standards were used for calibration]. Detection was conducted using a differential refractive index detector, a light scattering detector operating with two angles at 90° and 15°, and a differential viscometer. *M*_n_, *M*_w_, and dispersities (*Đ* = *M*_w_/*M*_n_) were calculated on the basis of the PMMA calibration curve using the Agilent GPC/SEC software.

#### Inelastic neutron scattering

INS was performed at the VISION beamline (BL-16B) of the Spallation Neutron Source at Oak Ridge National Laboratory. The sample was treated in an inert atmosphere at 120°C for 2 hours to remove adsorbed water, followed by evacuation of the chamber. Next, the cell was cooled down to −268°C for spectra collection. DFT calculations were performed using the Vienna Ab initio Simulation Package (*64*). The calculation used projector-augmented wave method (*65*, *66*) to describe the effects of core electrons and Perdew-Burke-Ernzerhof (*67*) implementation of the generalized gradient approximation for the exchange-correlation functional. The energy cutoff was 800 eV for the plane-wave basis of the valence electrons. The lattice parameters and atomic coordinates from literature (*68*) were used as the initial structure. Electronic structure was calculated on a Γ-centered 3 × 3 × 6 mesh. The total energy tolerance for electronic energy minimization and structure optimization was 10^−8^ and 10^−7^ eV, respectively. The maximum interatomic force after relaxation was below 0.001 eV/Å. The optB86b-vdW functional (*69*) for dispersion corrections was applied. The interatomic force constants were calculated by density functional perturbation theory on a 1 × 2 × 2 supercell, and the vibrational eigenfrequencies and modes were then calculated using Phonopy (*70*). The OCLIMAX software (*62*) was used to convert the DFT-calculated phonon results to the simulated INS spectra.

#### X-ray absorption spectroscopy

XAS was performed ex situ in the beamline 7-BM of the National Synchrotron Light Source II at Brookhaven National Laboratory. Note that the presented Ga K edge and In K edge XAS data for fresh LM, spent LM, and carbonaceous product were collected in fluorescence mode. For the Ga K edge data of fresh and spent Ga LM (and In-Ga LM) self-absorption effects are expected. For Ga remaining in the carbonaceous products obtained using Ga and In-Ga LMs and for In in fresh and spent In-Ga LM samples, no self-absorption effects were observed.
