# Supplementary material for: Low-temperature dechlorination of polyvinyl chloride (PVC) for production of H2 and carbon materials using liquid metal catalysts
Source: Sci Adv. 2024 Jul 24;10(30):eadm9963. doi: 10.1126/sciadv.adm9963 (PMC11268417; doi:10.1126/sciadv.adm9963)
Supplement: Supplementary file 1 — Tables S1 to S3 Figs. S1 to S11 [file sciadv.adm9963_sm.pdf]

Science Advances
AAAS

Supplementary Materials for

Low-temperature dechlorination of polyvinyl chloride (PVC) for production of H_2 and carbon materials using liquid metal catalysts

Felipe Polo-Garzon *et al.*

Corresponding author: Felipe Polo-Garzon, pologarzonf@ornl.gov

Sci. Adv. **10**, eadm9963 (2024)
DOI: 10.1126/sciadv.adm9963

This PDF file includes:

Tables S1 to S3
Figs. S1 to S11

Table S1 Comparison between state-of-art of PVC conversion and this work.

Feature	State-of-art from literature*				This work**
**Reactant**	PVC	PVC	PVC/ PET	PVC/PP	PVC
**Refs**	Ling, M. *et al., Chemosphere* **2023**, 316, 137718. *(4)*<br><br>Svadlenak, S. *et al.*, *Appl. Catal. B: Environ.* **2023**, *338*, 123065. *(8)*	Feng, B. *et al., Appl. Catal. B: Environ.* **2023,** *331*, 122671. *(13)*	Cao, R. *et al.*, *Nat. Sustain.* **2023,** *6*, 1685-1692. *(14)*	Kots, P. A. *et al.*, *Nat. Sustain.* **2023**, *6*, 1258-1267. *(9)*	
**Product**	Hydrochar, long hydrocarbons	Organic chloride, PE-like polymer	TPA, EDC, DHPVC	Long hydrocarbons	Cyclic aliphatic polymer
**T (°C)**	200-300	180	130-230	250	200
**Catalyst, absorbent, solvent**	Alkaline additives, H_2O, Pt/C, Pd/C	Ru/Al_2O_3, THF	Bu_4PCl (IL) + $ZnCl_2$	$Mg_3AlO_{4.5}$, Ru/TiO_2	Ga LM
**Cl removal (%)**	> 90	~100	> 95	> 90	~90
**H_2 yield (%)**	0	0	N/A	0	11
**Other operational parameters/ characteristics**	~300 psi [*Chemosphere* **2023**]. H_2 co-feed (94 psi)[*Appl. Catal. B: Environ.* **2023**]. Cl^- sequestrated in a salt	H_2 co-feed (290 psi)	DHPVC is an unused by-product	H_2 co-feed (435 psi). Cl^- sequestrated in a salt	

** PE= polyethylene, PP= polypropylene, THF= tetrahydrofuran, TPA= Terephthalic acid, EDC= 1,2-dichloroethane, DHPVC= partially dechlorinated polyene, IL= ionic liquid.*

***Cl^- is entrapped in carbon product and removed in a postreaction acetone-wash at room temperature.*

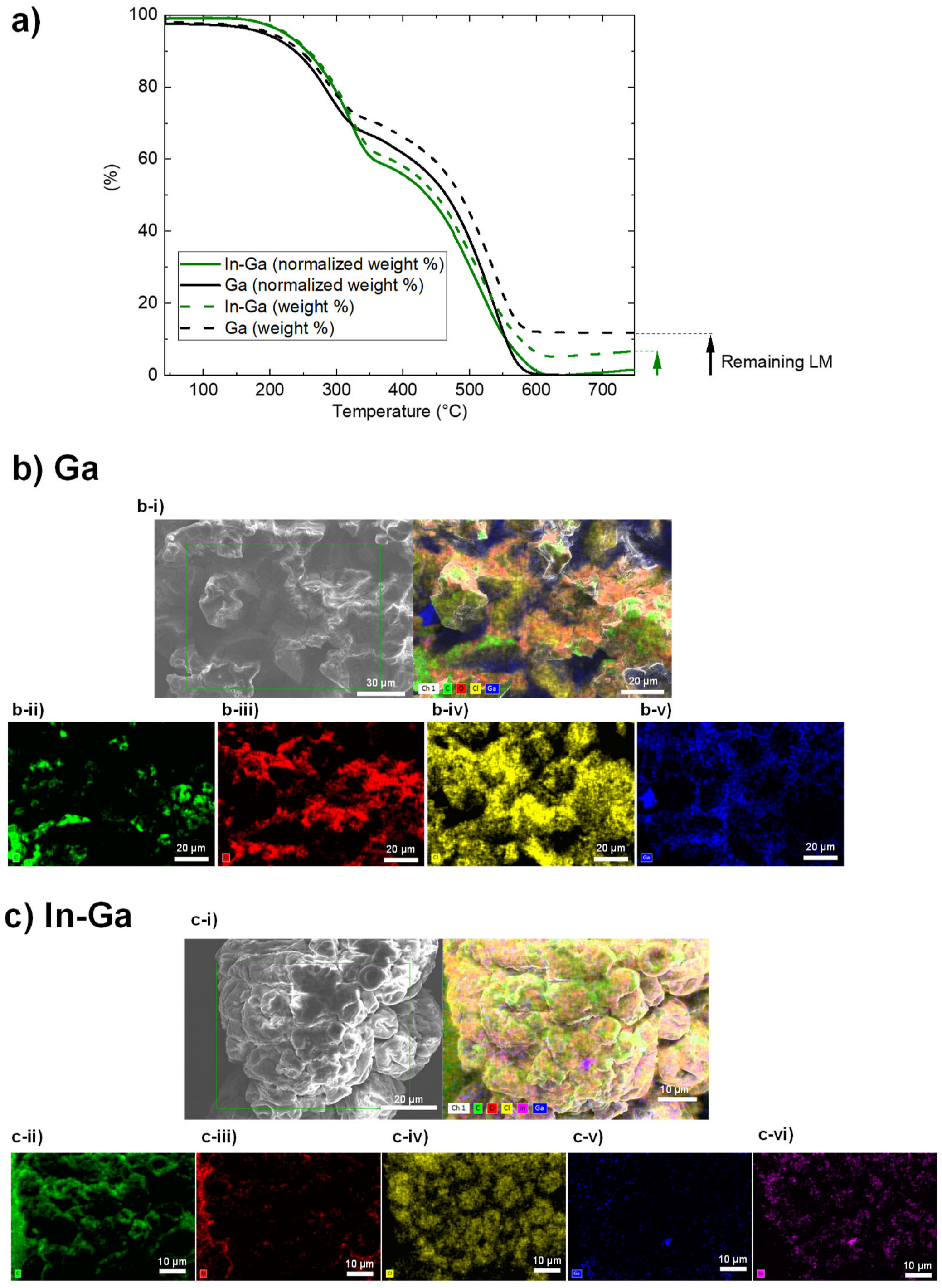

Fig. S1 Carbonaceous products obtained from conducting the batch reaction under Ar atmosphere at 200 °C for 1 h using 5 g of LM and 0.2 g of PVC. a) TGA in air. **b-c)** EDS mapping: i) all-element mapping, ii) C, iii) O, iv) Cl, v) Ga, vi) In.

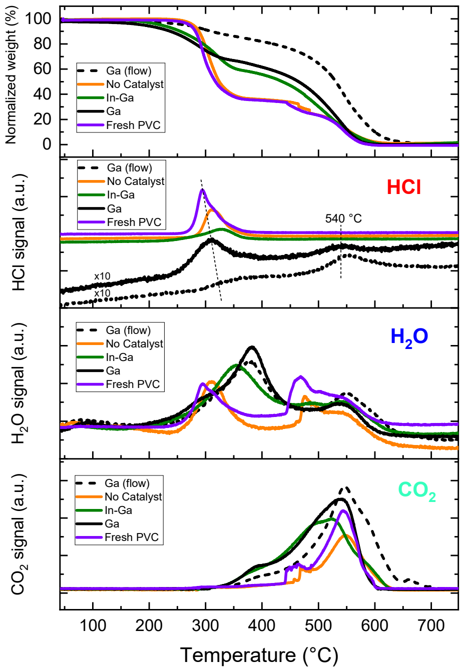

Fig. S2 TGA-MS in air. Fresh PVC and carbonaceous products obtained from conducting the reaction under Ar atmosphere at 200 °C for 1 h using 5 g of LM (or without LM) and 0.2 g of PVC. Batch mode: 'Ga', 'In-Ga', 'No Catalyst'. Flow mode: 'Ga (flow)'. Refer to **Fig. 1** for more details.

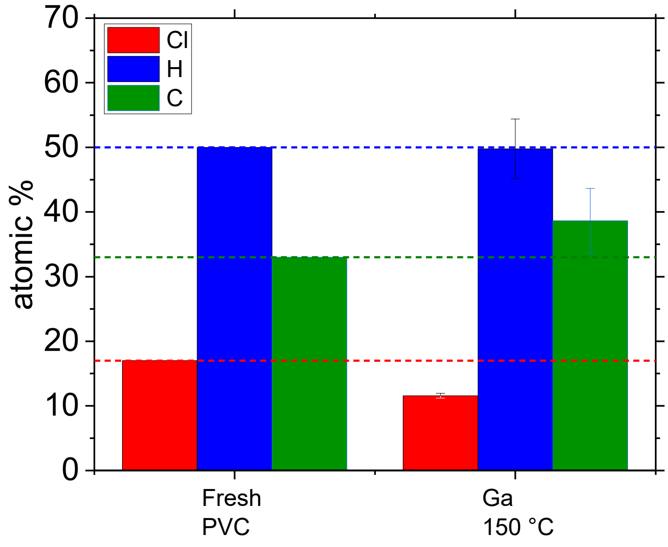

Fig. S3 Atomic composition of fresh PVC and carbonaceous product. Reaction was conducted under Ar atmosphere at 150 °C for 1 h using 5 g of Ga and 0.2 g of PVC (batch mode).

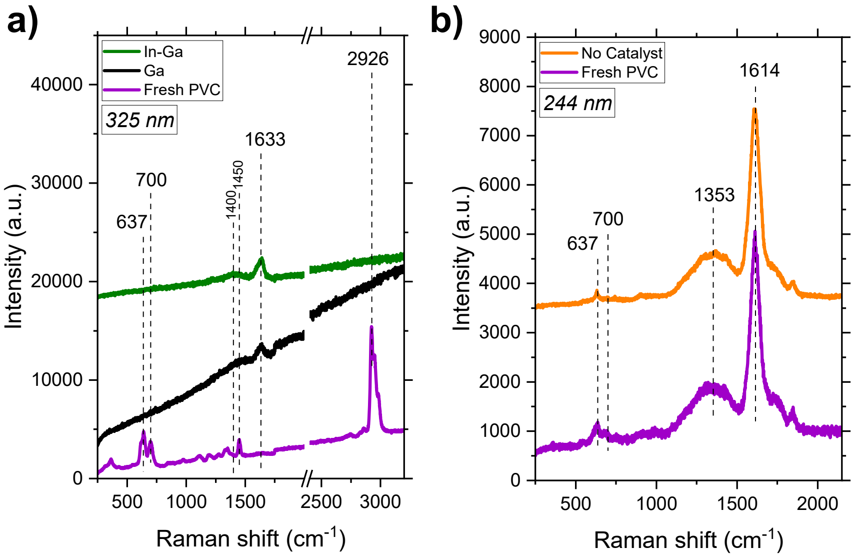

Fig. S4 Raman spectroscopy. a) 325 nm and **b)** 244 nm laser excitation. Fresh PVC and carbonaceous product after batch reaction with catalyst ("Ga", "In-Ga") and without catalyst ("No Catalyst") at 200 °C, 5 g LM, 0.2 g PVC, 1h, batch mode. *Peaks at 1353 and 1614 cm^{-1} in b) arise from burning the sample at 244 nm excitation. This excitation was used to study the "No Catalyst" sample as other excitations only provided strong fluorescence background.*

XPS results

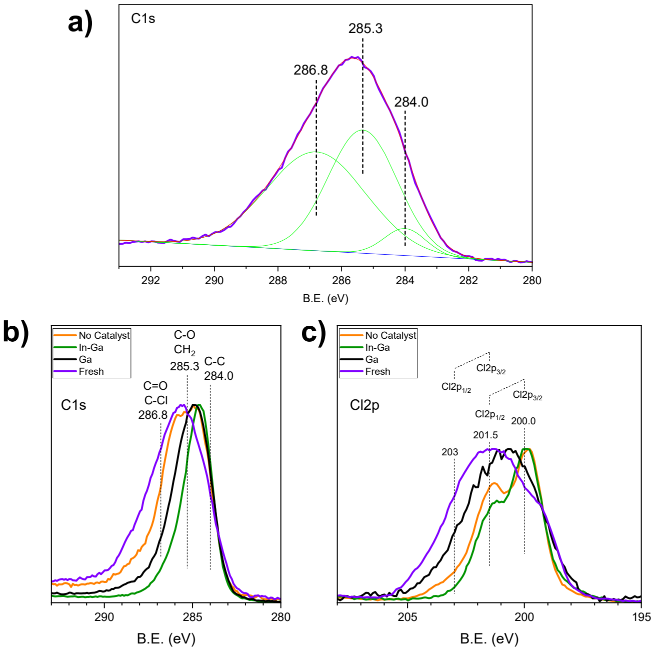

Fig. S5 XPS characterization. a) XPS spectra of C 1s of Fresh PVC. XPS spectra of **b)** C1s and **d)** Cl2p for Fresh PVC and carbonaceous product after batch reaction with catalyst ("Ga", "In-Ga") and without catalyst ("No Catalyst") at 200 °C, 5 g LM, 0.2 g PVC, 1h, batch mode.

Analysis of XPS spectra revealed that there are three types of carbon atoms present in the fresh PVC, with binding energies (BE) at approximately 286.8, 285.3 and 284.0 eV, attributed to C-Cl (or C=O), CH_2 (or C-O), and C-C, respectively (**Fig.**

S5a). The C-C bond could potentially be attributed to unsaturated C=C bonds. Despite the overlapping binding energy for C-Cl and C=O, and for CH_2 and C-O, the changes in the spectra generally support the dechlorination observed via TGA-MS analysis. Compared with the fresh PVC sample, the thermally treated sample underwent a partial reduction of the C-Cl signal, and an increase in the C-C signal, suggesting some dechlorination and generation of unsaturated double bonds. However, the spectra for the carbonaceous products obtained via LM catalysis are dominated by the C-C feature, with the C-Cl and CH_2 features significantly reduced. The C-C feature in the carbonaceous products after LM catalysis could be due to unsaturated C=C bonds or newly formed C-C bonds. The change in the shape of Cl2p core level spectra for carbonaceous products compared to fresh PVC reveals that at least two types of Cl bonds are present (**Fig. S5c**). Two sets of doublets can be identified, the first one with the $Cl2p_{3/2}$ centered at ~ 200 eV, and a second one with the $Cl2p_{3/2}$ centered at ~201.5 eV. Thus, the $Cl2p_{1/2}$ of the first doublet overlaps the $Cl2p_{3/2}$ of the second doublet. The catalytic and thermal treatment appear to preferentially remove the Cl associated with the second doublet ($Cl2p_{3/2}$ at ~201.5 eV); however, further investigations are needed to comprehend the coordination environment of the Cl atoms present in the structure.

IR spectroscopy

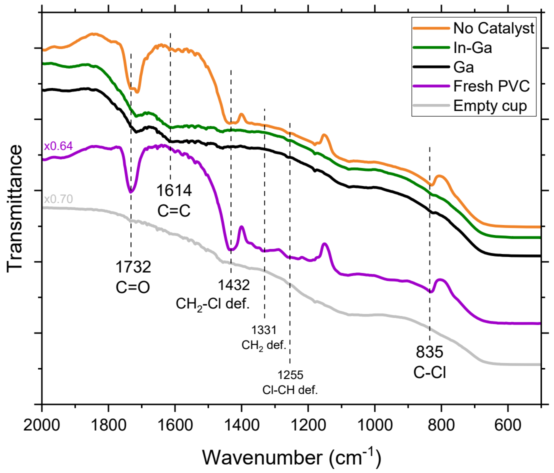

Fig. S6 DRIFTS. Fresh PVC and carbonaceous product after batch reaction with catalyst (“Ga”, “In-Ga”) and without catalyst (“No Catalyst”) at 200 °C, 5 g LM, 0.2 g PVC, 1h, batch mode (spectrum without a sample (“Empty cup”) is included as reference).

The band at 835 cm^{-1}, associated with the C-Cl stretching in pure PVC, is absent for the carbonaceous product after conversion using LM catalysts; however, it remains when PVC was thermally treated in the absence of a catalyst. CH_2 group deformation (1331 cm^{-1}) and Cl-CH out-of-plane angular deformation (1255 cm^{-1}), are observed in fresh PVC, but their intensities are insignificant for the samples treated at 200 °C (with or without catalyst).

Although present in fresh PVC and heat-treated PVC, CH_2-Cl angular deformation (1432 cm^{-1}) is not present in the carbonaceous products obtained via LM catalysis. These vibrational signatures suggest the creation of a more rigid and dechlorinated structure for the LM-catalyzed conversion. The carbonaceous products obtained in the LM-catalyzed process, show a vibrational mode at 1614 cm^{-1}, ascribed to C=C bonds, which is absent in pure PVC. The creation of these double bonds is confirmed via ^{13}C MAS NMR (see **Fig. 5a**), although their relative concentration is minor. The band at 1732 cm^{-1} is attributed to C=O stretching. This feature considerably reduces in the carbonaceous products obtained using Ga or In-Ga catalysts.

Table S2 Assignment of vibrational modes of simulated INS spectra in Fig. 6.

Wavenumber (cm^{-1})		Vibration type and contribution (major, minor, or similar)								
		-CH$_2$- angle change				-CH$_2$- bond-length change		-CHCl-		
		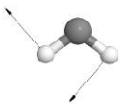	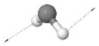	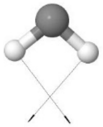	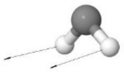	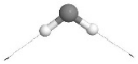	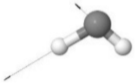	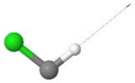	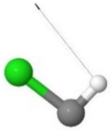	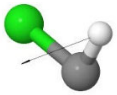
		-CH$_2$- rocking	-CH$_2$- twisting	-CH$_2$- bending	-CH$_2$- wagging	-CH$_2$- symmetrical stretching	-CH$_2$- asymmetrical stretching	H stretching in -CHCl-	H rocking in -CHCl-	H wagging in -CHCl-
1024-2927	1024		minor							major
	1045, 1047	similar			similar				similar	
	1077, 1078				major					minor
	1089		minor						major	
	1160		major							minor
	1162, 1165		minor						major	
	1202-1203				minor					major
	1224, 1226								major	
	1301		minor							major
	1309, 1315		similar						similar	
	1333				major				minor	
	1357-1358				similar					similar
	1386, 1388, 1404, 1406			major						
2963-2997	2920, 2926, 2927					major				
	2963						major			
	2965						similar	similar		
	2992							major		
	2996-2997						minor	major		

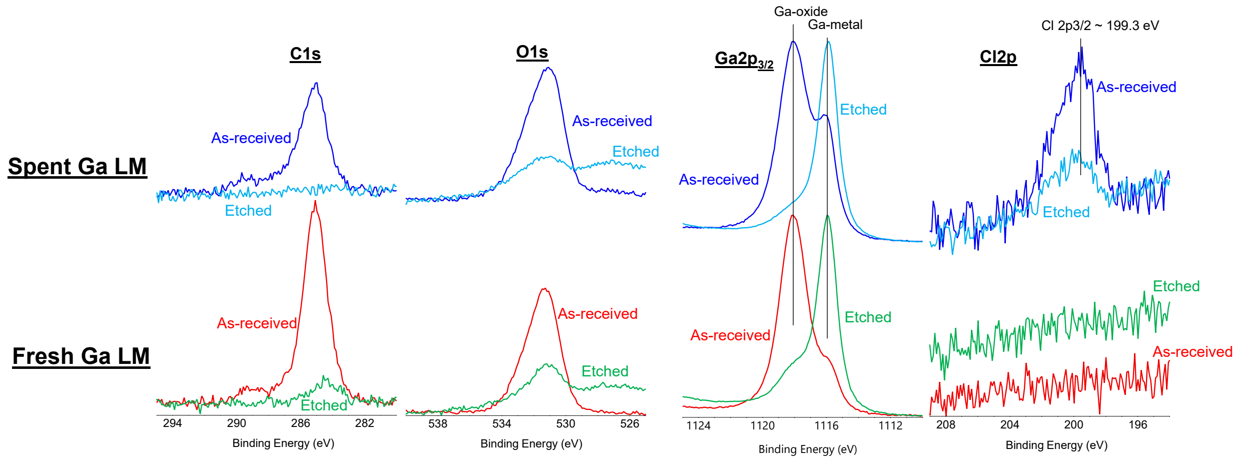

Fig. S7 XPS characterization. Spectra of C1s, O1s, Ga2p3/2 and Cl2p for Fresh Ga LM and Spent Ga LM after reaction at 200 °C (5 g Ga, 0.2 g PVC, 1h, batch mode). Spectra collected on as-received samples and on etched samples. Etched samples were generated after Ar-ion depth profiling for ~8 min.

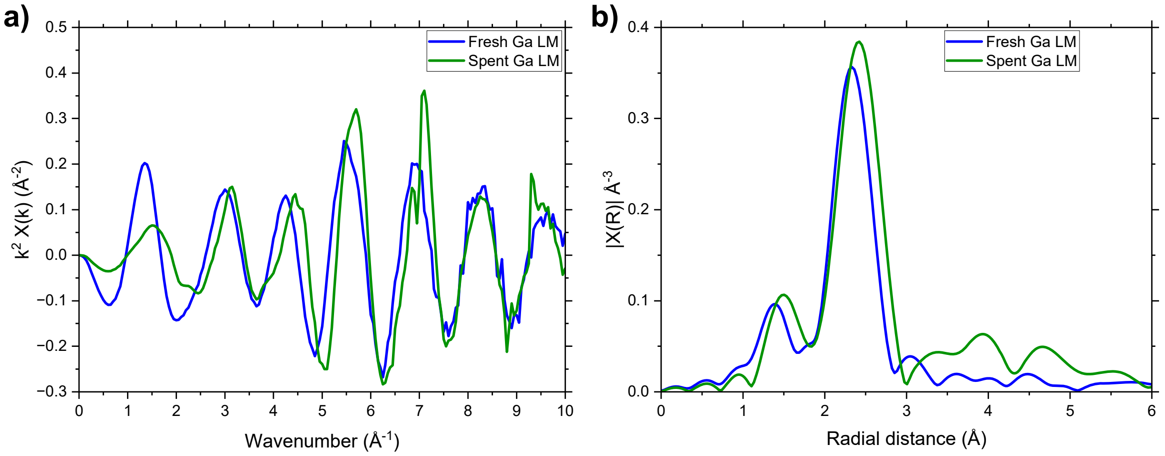

Fig. S8 EXAFS of Ga LM catalyst. a) k space and **b)** R space EXAFS data of fresh and spent Ga LM catalyst. Reaction conditions: batch mode, Ar atmosphere, 200 °C, 1 h, 0.2 g of Fresh PVC.

Table S3 Ga K edge and In K edge EXAFS and XANES fitting for fresh LM, spent LM, and carbonaceous product.

Sample	Condition	Path	R (Å)	ΔE₀ (eV)
**Ga LM catalyst**	Fresh	Ga-Ga	2.42±0.06<br>2.67±0.02	-4.4±1.7<br>-4.4±1.7
	Spent	Ga-Ga	2.51±0.06<br>2.71±0.02	3.2±2.2<br>3.2±2.2
**Carbon product**	Obtained using Ga LM catalyst	Ga-Cl	2.14±0.01	-0.2±1.4
**Carbon product**	Obtained using In-Ga LM catalyst	Ga-Cl<br>In-Cl	2.172±0.008<br>2.37±0.01	6.1±1.1<br>-3.4±1.2
**Ga_2O_3**		Ga-O	1.91±0.03	8.8±2.8
**In_2O_3**		In-O	2.17±0.01	4.5±1.2

Reaction conditions: batch mode, Ar atmosphere, 200 °C, 1 h, 5 g LM, 0.2 g of Fresh PVC.

For the fresh and spent Ga LM catalysts, the fitting model includes two Ga-Ga paths since based on the crystal structure of metal Ga (space group: C m c a, a=4.519Å, b=7.658Å, c=4,562Å, α=ß=γ=90°), the first nearest Ga-Ga bond distances range from 2.48 to 2.78 Å. [von Schnering, H. G.; Nesper, R., *Acta Chem. Scand.* **1991**, *45*, 870-872.] Due to the self-absorption effects, for these two samples, we only aim to obtain quantitative information on Ga-Ga bond distances. The fitting k range is 2-10$Å^{-1}$ and the fitting R range is 1.5-2.9Å. For Ga (In) data of carbon products, two fitting models are tested: one includes Ga (In)-O path and the other includes Ga(In)-Cl path. The best fitting results can only be obtained by using Ga(In)-Cl path. Using Ga(In)-O, large threshold energy shift (ΔE0) is obtained (>15 eV). In fitting the spectra of Ga (In) of carbon products, the fitting k range is 2-11$Å^{-1}$ and the fitting R range is 1.0-2.2Å.

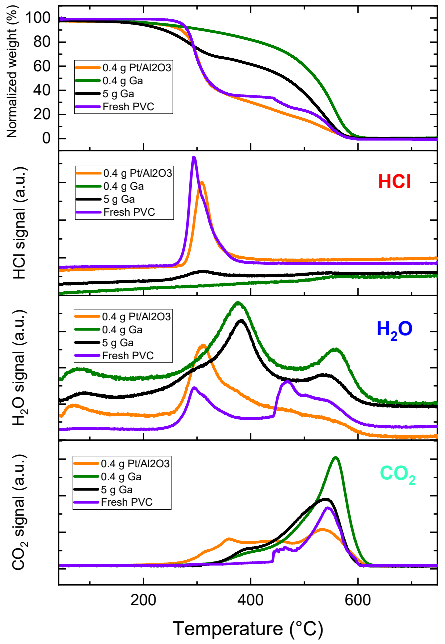

Fig. S9 TGA-MS in air. Fresh PVC and carbonaceous products obtained after conducting the reaction in batch mode under Ar atmosphere at 200 °C for 1 h using 0.2 g of PVC and a catalyst.

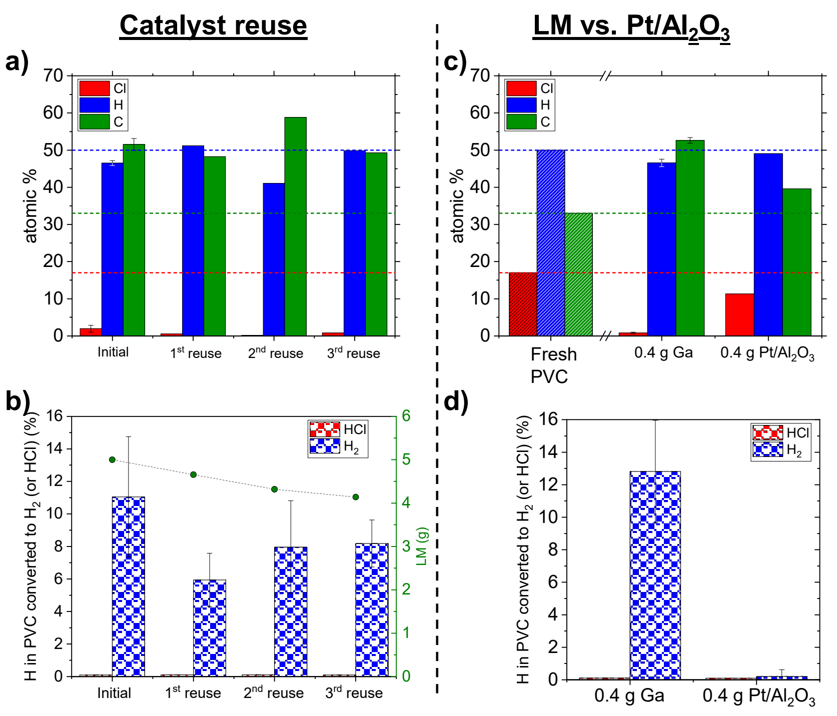

Fig. S10 Catalyst reuse and comparison to traditional solid catalyst. a-b) Test of catalyst reuse using initial 5 g of Ga LM catalyst, each reuse is performed using 0.2 g of PVC (Cl/Ga molar ratio=0.04): **a)** atomic composition of carbonaceous products, and **b)** gas H_2 and HCl product distribution.
c-d) LM vs. traditional solid catalyst Pt/Al_2O_3: **c)** atomic composition of carbonaceous products, and **d)** gas H_2 and HCl product distribution. Reaction was conducted in batch mode under Ar atmosphere at 200 °C for 1 h using 0.2 g of PVC and a catalyst.

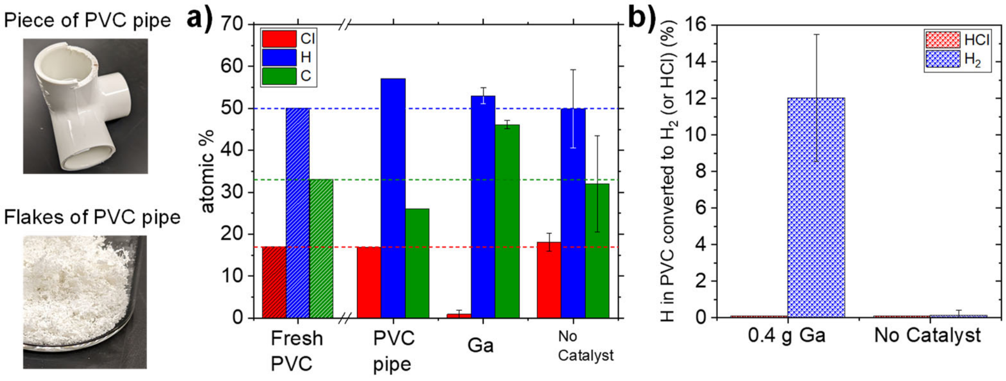

Fig. S 11 Conversion of discarded PVC pipe. a) Atomic composition of Fresh PVC, PVC pipe, and carbonaceous products obtained after catalytic (0.4 g of Ga) and non-catalytic conversion of PVC pipe, **b)** Gas phase product distribution. Reaction was conducted in batch mode under Ar atmosphere at 200 °C for 1 h using 0.2 g of discarded PVC pipe (shredded into flakes).
